# The Association between the Binding Processes of Working Memory and Vascular Risk Profile in Adults

**DOI:** 10.3390/brainsci11091140

**Published:** 2021-08-28

**Authors:** Eirini Bika, Despina Moraitou, Elvira Masoura, George Kolios, Georgia Papantoniou, Maria Sofologi, Vasileios Papaliagkas, Georgios Ntritsos

**Affiliations:** 1 Laboratory of Psychology, Section of Experimental and Cognitive Psychology, School of Psychology, Faculty of Philosophy, Aristotle University of Thessaloniki, 54124 Thessaloniki, Greece; demorait@psy.auth.gr (D.M.); emasoura@psy.auth.gr (E.M.); 2 Laboratory of Neurodegenerative Diseases, Center for Interdisciplinary Research and Innovation (CIRI-AUTH), Balkan Center, Aristotle University of Thessaloniki, Buildings A & B, 10th km Thessaloniki-Thermi Rd, P.O. Box 8318, 57001 Thessaloniki, Greece; gpapanto@uoi.gr; 3 IT of the School of Psychology, Aristotle University of Thessaloniki, 54124 Thessaloniki, Greece; georgekolios@psy.auth.gr; 4 Laboratory of Psychology, Department of Early Childhood Education, School of Education, University of Ioannina, 45110 Ioannina, Greece; m.sofologi@uoi.gr; 5 Institute of Humanities and Social Sciences, University Research Centre of Ioannina (U.R.C.I.), 45100 Ioannina, Greece; 6 Department of Biomedical Sciences, Alexander Campus, International Hellenic University, P.O. Box 141, Sindos, 57400 Thessaloniki, Greece; vpapaliagkas@mls.teithe.gr; 7 Department of Hygiene and Epidemiology, University of Ioannina Medical School, 45110 Ioannina, Greece; gntritsos@uoi.gr; 8 Department of Informatics and Telecommunications, School of Informatics and Telecommunications, University of Ioannina, 47150 Arta, Greece

**Keywords:** working memory (WM), episodic buffer (EB), feature binding test (FBT), computerized tool, vascular risk profile (VRP)

## Abstract

Episodic buffer (EB), a key component of working memory, seems to have a rather complicated function as part of binding processes. Recent papers on the field claim that binding processes of working memory (WM) are assisted by attention and executive functions. On the same page, vascular pathology is gaining more ground as the main underlying cause for many brain pathologies. Hypercholesterolemia, hypertension, obesity, diabetes, lack of exercise and smoking are the most common risk factors that people of all ages suffer from and constitute the main vascular risk factors responsible for a possible decline in executive functions and attention. Thus, this research is an attempt to examine the relation between the binding functions of WM and the existence of vascular risk factors via a computerized test focusing on feature binding. The study comprised adults (n = 229) with and without vascular risk factors. The main tools used were a biomarker questionnaire and a feature binding test (FBT). The results showed that participants who report suffering from one or more vascular risk factors had significantly lower performance on specific subtasks of the FBT in comparison to the participants who were healthy. This allows us to assume that there might be a positive association between feature binding and a vascular risk profile in adults, and such a test could be a useful diagnostic tool for early cognitive impairment due to incipient vascular pathology.

## 1. Introduction

“Working memory (WM)” is a rather elusive concept with many questions about its function and structure still unanswered [1,2]. The most widely accepted and proved model of WM is the multicomponent model of WM, which consists of the central executive (CE) (attention control), the phonological loop (responsible for the retention of phonologic/auditory information) and the visuospatial sketchpad (responsible for the retention of visual (shape) and spatial (location) information) [3]. Baddeley and Logie [4] noticed that CE was incapable of coordinating the two subsystems (phonological loop and visuospatial sketchpad), controlling attention switching and activating long-term memory presentations at the same time, due to its limited capacity. “Episodic buffer” (EB) was added then, as a component that supports and assists the functions of CE, providing it with the necessary capacity for its functions and coordinating the other subsystems in order to bind all the different information in one episode/chunk [4,5,6]. As EB is the latest addition to the multicomponent model of WM, many questions about its function, capacity and nature have arisen.

In an attempt to study this component of WM under the spectrum of the current developments in the field of brain pathologies and cognitive decline, we decided to examine if there are any possible associations between its functions and vascular pathology. Prompted by papers supporting that there is a common vascular pathologic background in almost all dementia conditions [7,8,9,10,11,12], we assumed that vascular risk factors, such as hypertension, hypercholesterolemia, smoking, lack of exercise and obesity, may also affect EB’s functions.

There are many methods to extract and analyze data from how the brain works. Brain–computer interfaces, electroencephalogram (EGG) and functional magnetic resonance imaging (FMRI) are some of the updated ways [13]. In the same direction, we tried to develop a new computerized tool able to incorporate the various tasks needed for the detection of the cognitive functions that we study here.

### 1.1. Episodic Buffer and Binding Processes

Episodic buffer is a limited-capacity system that provides short-term storage of information formed in a multimodal mode, which can bind information from the subsystems of working memory and from long-term memory into a unitary “episode” oriented in time and place, using executive functions [5]. The addition of this component is an attempt to handle the more complicated aspects of cognitive control in WM. The process of feature binding seems to require the activation of some executive functions, and especially of the executive control, attention and fluid intelligence [14]. Many researchers tend to support that EB is similar to the focus of attention described by Cowan [15], as it is used to describe the active storage of accessible representations [16]. However, more recent papers suggest that it is a rather passive system, but with a crucial role in integrating information from different dimensions into utilized episodes or chunks [17,18]. Specifically, it seems to be capable of storing bound features and making them available to conscious awareness, but it is not itself responsible for the process of binding [19,20]. 

Later studies on the WM and the binding processes of EB seem to agree that both notions are in part accurate. Allen, Baddeley and Hitch [21], after conducting a series of experiments in serial WM, concluded that there are two components in serial visual WM: one that automatically saves the last item presented within the focus of attention and thus is susceptible to executive disruption and one that activates executive functions in order to maintain the representation on the focus of attention. 

On the same page, in this study, we decided to focus on feature binding, as it depends, mainly, on the most complex system in WM (visual WM), and it seems to relate to the function of EB in general [17]. It also provides us with important information about the role of attention processes in WM [21,22], and thus, it is more useful for our purposes.

### 1.2. Visual Binding, Executive Functions, and Attentional Control

Binding in short-term memory refers to the grouping of an object’s features, such as shape, color or location, so that they form a unitary object distinct from others in memory [17,23]. Baddeley [5] suggested that feature binding takes place mainly within EB and is highly dependent on executive-based attentional control. Speculating on this, Wheeler and Treisman [24] supported that focused attention contributes to the maintenance of bound object representations in WM and that when attention is withdrawn or disrupted, they disappear. Hitch, Allen and Baddeley [22] also claim that attention plays a critical role in encoding and maintaining bound representations in WM, but into account of CE. Moreover, they concluded that there are two forms of attention in visual binding, the internal (executive) and the external (perceptual) considering the different storage capacity, that are supplementary and contribute to the feature binding [22].

There are many controversies when considering the role of attention in binding processes, especially in visual WM. In an effort to resolve these inconsistent results, Allen, Hitch, Mate and Baddeley [25] conducted a series of experiments on feature binding and attention. The findings revealed that remembering feature bindings is no more attention-demanding than recalling individual features and that binding takes place automatically within the visuospatial sketchpad, which they regarded as a multilevel store holding information about both feature and object levels. They supported that information from the visuospatial sketchpad was accessible to EB only for conscious control and manipulation [25]. In other words, they support that perceptual and executive attention works in a different but complementary way in the process of binding [25,26]. 

### 1.3. Vascular Risk Factors, Executive Functions and Attention

It is strongly supported that the existence of vascular risk factors is associated with the dysfunction of executive functions, constituting various brain pathologies such as vascular cognitive impairment and vascular dementia [7,10,27,28,29,30,31].

Hypertension seems to affect cognitive functions due to alterations in the blood flow and brain connectivity, which results in lessened white matter [32,33]. Executive functions, motor and processing speed, working memory and attention appear to be worsened in people dealing with hypertension [33,34,35].

On the same page, smoking, due to nicotine, which binds to a wide range of acetylcholine receptors, influences a wide variety of cognitive domains, such as sensorial, motor, attention, executive function, learning and memory [36]. Obesity and overweight seem to be associated with a general executive dysfunction, but direct effects on specific functions are unable to be distinguished [37].

A vast number of studies have been conducted in order to examine the effects of exercise on executive functions. Most of them support that medium-intensity aerobic exercise subserves executive functions as it is responsible for many structural and neural changes, and this is the main reason that it is highly recommended, especially for older people or people suffering from cognitive impairment [38,39,40,41]. In addition to this, physical activity acts beneficially on other vascular risk factors (e.g., type 2 diabetes, hypertension, obesity), by reducing the risk of harm and improving cognition [42]. Diamond [43] supports that the lack of exercise is associated with impaired executive functions.

Moreover, patients who suffer from type 1 and type 2 diabetes mellitus experience dysfunction in executive functions, attention and memory [44,45]. Taking into consideration the newest findings in this field, which support that Alzheimer’s may also be a new type of diabetes [46,47], it becomes clear that there is a strong association between high blood sugar and executive dysfunction [48]. Last but not least, there seem to be some incoherent associations between the levels of low-density lipoprotein (LDL) cholesterol and cognitive functions; these incoherencies may be due to the different ages and domains of function focused on in each study [49,50].

Considering the above findings, it becomes clear that vascular factors are associated with executive dysfunctions. Combining this notion with the fact that feature binding processes are related to executive functions, we presume that executive functions may be impaired in some way in individuals who suffer from one or more factors, and this may be the very first sign of cognitive impairment.

### 1.4. The Purpose and the Hypotheses of the Study

Based on the theoretical background, the aim of this study was to examine whether there is a relation between the binding processes in visual WM and a series of self-referred vascular risk factors. Specifically, it tries to investigate whether the vascular pathologies that can affect executive functions also affect the visual binding abilities. Taking into account what has been mentioned before, the hypotheses of the study were formulated as follows: Participants who fit the “vascular risk profile” (VRP), i.e., suffer from one or more vascular risk factors, would show lower performance in a feature binding test compared to participants who do not suffer from any vascular risk factor, due to possible brain pathology accelerated by these factors (Hypothesis 1). Participants’ performance may be associated with the existence of specific vascular factors (e.g., hypertension, hypercholesterolemia, diabetes, smoking) or a specific combination of vascular factors, due to the way each of them may affect executive functions (Hypothesis 2).

## 2. Materials and Methods

### 2.1. Participants

The convenience sample comprised a total of 229 adults (29.3% males), Greek native speakers. Their age ranged from 18 to 75 years (M = 42.72, S.D. = 16.21), and they were divided into two groups: young adults, aged from 18 to 35 years old, and adults, aged from 36 to 75 years old. Most of them were of high educational level (62.9%) and lived with someone else (84.7%). Only 22.7% of participants did not meet the criteria for having a vascular risk profile, 32.3% had at least one vascular risk factor (VRF), 27.5% had at least two VRFs and 17.5% had more than three VRFs. More specifically, 39.3% did not exercise, 70.7% were overweight, 22.7% were regular smokers, 23.6% suffered from hypertension (21% were taking antihypertensive medication), 27.1% suffered from hypercholesterolemia (14.4% were taking cholesterol-lowering medication) and 5.7% suffered from diabetes (5.2% were taking antidiabetic medication) (Table 1). 

### 2.2. Tools

#### 2.2.1. Biomarker and Demographic Data Questionnaire

This questionnaire was created to collect all the essential information about the health history of the participants. Specifically, it was a self-reference questionnaire that consisted of 10 closed-ended questions about the existence or not of each vascular factor and 3 closed-ended questions about medication related to the vascular pathology (e.g., hypertension drugs). Participants were supposed to answer choosing between “Yes” or “No” (e.g., Do you smoke? “Do you suffer from hypertension?”) (Table 1). According to DSM-5, the occurrence of cerebrovascular risk factors responsible for neurodegenerative diseases could also be validated through the administration of health history without physical examination [51].

#### 2.2.2. Assessment of Episodic Buffer—Feature Binding Test (FBT)

In order to assess the function of EB of working memory, we created a tool that examines the binding processes based on a tool developed by Masoura and Pope [52]. Particularly, Masoura and Pope [53], in their attempt to examine the association between WM and bilingualism, created an integrating feature binding test in immediate memory, based closely on visual binding tasks suggested by Allen, Baddeley and Hitch [17] and the cross-modal binding task suggested by Quinette et al. [54]. Thus, the current tool is basically an adaptation of this test. The fact that it consists of both visual binding tasks (combination of shape, color and location—visuospatial sketchpad) and a multimodal binding task (letter and location—combination of functions of phonological loop and visuospatial sketchpad) assists in detecting differences in the temporary maintenance in both subsystems of WM.

It consists of three tasks. The first one is a visual binding task (VBT1) in which participants have to encode, retain and recall the color–shape combination of the objects presented. The second one is also a visual binding task (VBT2) but is more resource-demanding, as participants also have to remember the location of the object (color–shape–location). The third one is a multimodal binding task (MBT) focused on the memory load capacity to store multimodal information (verbal and spatial). All tasks are simple “Yes/No” recognition tasks and comprise 2 practice trials and 64 randomized test trials (8 × 8). The proportion of correct probes is exactly half (50%) in all tasks. The test was created and administered via an online electronic platform under the jurisdiction of A.U.Th (Laboratory of Psychology, School of Psychology).

**Figure 1:**
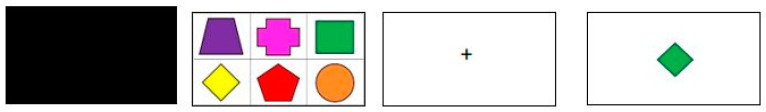
Picture sequence in VBT1.

Task 1 (Visual Binding Task 1 (VBT1)) is based on the color–shape combination condition in Allen’s classic task (2006). The color–shape combinations are created randomly from a pool of 8 colors (red, yellow, blue, pink, purple, brown, orange, green) and 8 shapes (square, cross, circle, pentagon, trapeze, rectangle, triangle, diamond). All items appear in a balanced amount. In the beginning, 6 colored shapes appear on the screen in a 3 × 3 grid for 2000 ms (2 s). The participants are instructed to remember the color–shape combinations. After a fixation interval of 2000 ms, during which a white screen with a black fixation cross in the center appears, one color–shape combination appears in the center of the screen, and just as in the task of Allen, Baddeley and Hitch [17], participants are instructed to judge whether the probe is one of the six in the original array. There is no time limit for the test’s recognition phase, and accuracy is emphasized over speed. Participants are instructed to select, using the computer mouse, the “True” or “False” button under the probe, according to whether the shape was presented earlier or not. After that, they move on to the next set of shapes (Figure 1).

**Figure 2:**
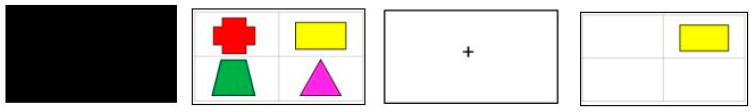
Picture sequence in VBT2.

Task 2 (VBT2) incorporates location in the binding process. Four colored shapes appear on the screen in a 2 × 2 grid for 2000 ms. Participants are instructed to remember the color–shape combinations. After the fixation cross (2000 ms), one color–shape combination appears on the screen, but instead of being in the center, the new probe appears within the same 2 × 2 grid, in one of the four squares. Participants are instructed to judge whether the singular probe is exactly the same as one of the original four, in color, shape and location (Figure 2). The rest of the instructions and layout stay the same as in VBT1. By adding the dimension of “location”, we tried to create a more demanding binding task that goes beyond just visual features, such as shape and color, and incorporates the spatial feature.

**Figure 3:**
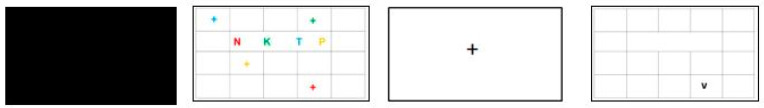
Picture sequence in MBT.

Task 3 (MBT) is a multimodal binding task in which verbal information was combined with spatial information, based closely on the cross-modal binding task of Quinette et al. [54], as derived from Mitchell et al. [23] and Prabhakaran et al. [52]. Firstly, 4 colored upper-case consonants (from the Greek alphabet, font Ariel, size 12) are displayed in the center of a 5 × 4 grid, and 4 colored crosses are placed randomly in the remaining 17 squares (green, blue, red and yellow). Participants are informed that each letter is matched with a location, as represented by the crosses, according to their common color, and are instructed to remember these pairs. Afterward, there is a fixation cross of 2000 ms, during which participants have to maintain the 4 associations. In the test phase, the same 5 × 4 grid is provided, with a single lower-case consonant (same as one of the previous 4 capital letters) presented in black color inside one of the squares. Participants are instructed to judge by selecting “True”/“False” buttons, using the computer mouse, if the location of the singular letter is correct depending on whether the matching is the same as before (Figure 3). As in the previous tests, there is no time limit in the test phase, and accuracy is emphasized over speed.

The score of each task is extracted by adding the correct answers. Each correct answer corresponds to a successful recognition and retrieval of the object presented. 

### 2.3. Procedure

As far as the administration is concerned, participants were informed of the aims and purposes of this study via social media platform (Facebook) for more direct responses. Since the tests are part of an electronic platform, people were able to participate from their own place using their own computer/tablet, serving the purposes of social distancing due to the ongoing pandemic (COVID-19). The length of the test administration was almost an hour. Explicit instructions were given to participants concerning the circumstances under which they were supposed to answer the tests (e.g., they should have been concentrated, well-rested, in a quiet place without distractions, with connection to the Internet and have plenty of time).

### 2.4. Ethics Statement

The authors assert that all procedures contributing to this work comply with the ethical standards of the relevant national and institutional committees on human experimentation. Specifically, all the participants were informed in writing of the purpose of the study. They were also informed that their data would be confidentially collected in an electronic database. The participants gave written informed consent, agreeing that their participation was voluntary and that they could withdraw at any time, without giving a reason and without cost. Due to the specific type of the current research, demographic data such as age, gender and personal medical history were collected. Since these are considered personal data, the European Union law that has existed since 28 May 2018 was applied. According to the law, the use of sensitive personal data is allowed only for research reasons. Therefore, the participants were informed accordingly, and they also agreed that their personal data could be deleted from the web database after a written request. The study’s protocol was approved by the Scientific and Ethics Committee of the School of Psychology, Aristotle University of Thessaloniki, and followed the principles outlined in the Helsinki Declaration 1975, as revised in 2008 [55].

### 2.5. Statistical Analysis

The data analysis was conducted in SPSS Statistics version 26 [56]. The analyses carried out were (a) repeated measures analysis of variance (**ANOVA**) and (b) multivariate analysis of variance (**MANOVA**). The aim of the **ANOVA** analysis was to determine that the participants’ performance differs in the three tasks of the FBT and thus that the three tasks measure different aspects of EB. The main aim of the **MANOVA** type of analysis was to compare participants’ performance in the FBT tool according to their age, vascular risk profile and educational level. **MANOVA** was also conducted to compare participants’ performance in the FBT tool considering each vascular risk factor alone. Levene’s test was used to assess the equality of variances, and Box’s test was used for the assessment of the equivalence of covariance matrices. Partial eta-squared (η_p_^2^) was used for the estimation of the effect size. The selection of **ANOVA** as the main analysis for our data was made considering the fact that this is a basic study of a newly presented tool, and we wanted to be able to extract some general conclusions about its ability to detect early cognitive deficits in people suffering from cardiovascular factors.

## 3. Results

### 3.1. Feature Binding Test: Performance Comparison between the Three Conditions

**Figure 4:**
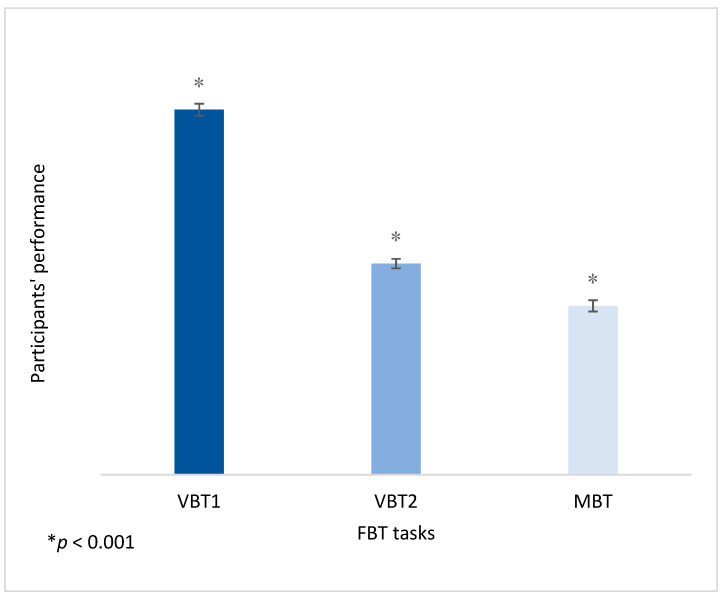
Participants’ performance in the three tasks of FBT.

In order to confirm that the three tasks of this tool differ significantly, repeated-measures **ANOVA** was conducted. The “FBT” was used as the factor that consisted of three levels (VBT1, VBT2 and MBT), in association with the three tasks of the tool. The participants’ scores in these three tasks were considered as the dependent variables. As Mauchly’s test of sphericity was statistically significant (*p* < 0.001), we accepted the Greenhouse–Geisser correction. Thus, the results showed that the performance in the three conditions of the FBT tool differed significantly (*F*(1.775, 248.568) = 474.003, *p* < 0.001, η_p_^2^ = 0.77). The Bonferroni pairwise comparisons test showed that participants’ performance was significantly higher (*p* < 0.001) in the VBT1 (*M* = 39.8369) compared to VBT2 (*M* = 23.0426; I-J = 16.794, *p* < 0.001) and MBT (*M* = 18.4326; I-J = 21.404, *p* < 0.001). Performance in VBT2 (*M* = 23.0426) was significantly higher than in the MBT (*M* = 18.4326; I-J = 4.610, *p* < 0.001) (Figure 4).

### 3.2. The Effects of Vascular Risk Profile on FBT Performance

When conducting **MANOVA**, we used the variables “age-group” (two levels: young adults, adults), “educational level” (measured in years of schooling (three levels: ≤6 years, 7–12 years, ≥13 years)) and “VRP” (four categories related to the number of vascular risk factors that each participant had: healthy, 1 factor, 2 factors, ≥3 factors) as independent variables. We considered participants’ scores in the three FBT tasks (“VBT1”, “VBT2”, “MBT”) as the dependent variables. As Box’s test for the assessment of the equivalence of covariance matrices was statistically significant (*p* < 0.05), we used Pillai’s trace. The results of the MANOVA showed a significant main effect of the VRP variable (Pillai’s trace(9, 354) = 0.246 *p* < 0.001, η_p_^2^ = 0.08) and age-group variable (Pillai’s trace(3, 116) = 0.072, *p* = 0.034, η_p_^2^ = 0.07). Moreover, **MANOVA** showed a significant interaction effect. Specifically, VRP × age-group marginally affected FBT performance (Pillai’s trace(9, 354) = 0.141, *p* = 0.046, η_p_^2^ = 0.05). 

**Figure 5:**
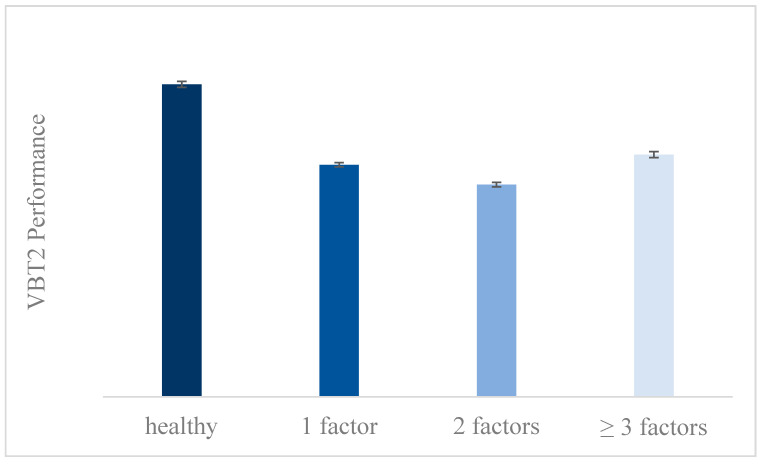
Participants’ performance in VBT2 according to their VRP.

In order to examine the specific effects of the independent variables and their interactions on each FBT task, we proceeded to the inspection of the results of each one of a series of ANOVAs. As Levene’s test for equality of variances was statistically significant for VBT2 and MBT (*p* < 0.05), only values that were of high statistical significance (*p* < 0.001) were accepted. The results showed a main effect of VRP only on VBT2 (*F*(3, 120) = 8.594, *p* < 0.001, η_p_^2^ = 0.18), with healthy participants showing a clear tendency of higher performance (*M* = 29.564) than participants who suffer from one (*M* = 21.944), two (*M* = 20.064) or three (*Μ* = 22.458) vascular risk factors (Figure 5). However, there was no statistically significant difference among them in pairwise comparisons. 

No statistically significant effect of age-group or VRP × age-group was found on the three FBT tasks (*p* > 0.05). 

### 3.3. VBT and Specific Vascular Risk Factors

In the subsequent **MANOVA**, we used the variables “exercise” (two levels: exercise, no exercise), “overweight” (two levels: normal weight, overweight–obesity), “smoking” (two levels: no smoking, smoking), “hypertension” (two levels: no hypertension, hypertension), “hypercholesterolemia” (two levels: no hypercholesterolemia, hypercholesterolemia) and “diabetes” (two levels: no diabetes, diabetes) as independent variables. As dependent variables, we considered participants’ scores in the three tasks of FBT (“VBT1”, “VBT2”, “MBT”). Since Box’s test for the assessment of the equivalence of covariance matrices was statistically significant (*p* < 0.001), we used Pillai’s trace. The results of the MANOVA showed no significant main effect of the specific vascular risk factors on participants’ performance in FBT. However, it was found that one type of interaction of independent variables significantly affected participants’ performance. Specifically, smoking × exercise (Pillai’s trace(3, 106) = 0.105, *p* = 0.008, η_p_^2^ = 0.10) was found to affect FBT performance. 

In order to examine the specific effects of the independent variables and their interactions on each FBT task, we proceeded to the inspection of the results of each one of a series of **ANOVA**s. As Levene’s test for equality of variances was statistically significant for VBT1 (*p* < 0.001) and MBT (*p* < 0.05), only values of high statistical significance were accepted. The inspection of the subsequent **ANOVA**s did not show any statistically significant effect. 

## 4. Discussion

The present study set out to investigate whether the existence of vascular risk factors is associated with the binding functions of WM, in an attempt to create and propose a new, alternative, computerized tool able to detect very early cognitive impairment due to incipient vascular pathology. According to our hypotheses, the VRP (existence of one or more than one vascular risk factor) and the type of the vascular factors (or of the combination of them) each participant suffered from would be associated with lower performance in the FBT tasks in comparison to participants who did not suffer from any vascular risk factors.

The three subtasks of the FBT differed significantly, affirming that they measure different aspects of binding processes and EB function and have an escalated difficultness in terms of attentional resources (first: color and shape features; second: color, shape and location features; third: verbal and spatial features). However, only the VBT2 managed to detect differences related to the VRP. This could have happened because VBT2 can depict the storage capacity of EB in a better way. In other words, VBT2, by incorporating both visual and spatial information, activates all the abilities of the visuospatial sketchpad, obtaining in this way stronger capacity and thus retaining a greater amount of information. On the contrary, VBT1, by incorporating only shape and color, activates only the visual part and therefore requires restricted capacity. In regards to the MBT task, the retaining of cross-domain features activates two different types of attention and shifting between them, giving it a higher level of difficultness even for young and healthy adults. Hence, it was found unable to detect slight differences between healthy people and people with a vascular risk profile, probably due to the “floor effects” condition that creates. This explains why participants, as a whole, had the lowest performance compared to the other two tasks.

The first hypothesis of this study was partially confirmed as the participants who fit the VRP and had one or more risk factors showed significantly lower performance in the FBT, especially in the VBT2, than healthy participants. According to the second hypothesis, the combination of smoking and lack of exercise seemed to be the one that was associated with problems in the binding processes. 

Our results about VRP seem to agree with the notion that people who suffer from vascular pathology may show deficits in working memory due to common executive dysfunction background. Recent papers on the field support that vascular pathology may be highly associated with cognitive impairment and especially executive dysfunction [8]. Accordingly, it is supported that in feature binding, the integration of color, shape and location or the integration of verbal information and location activates the frontal cortex, indicating the involvement of executive functions, in contrast to the single presentation of these features, which activates posterior brain areas [17,23,52]. Thus, it can be assumed that when people suffer from a vascular burden (have even one or more vascular risk factors), executive functions may show a tendency towards dysfunction [57].

However, when analyzing the results about the VRP and VBT2, we came across a paradox: participants who suffered from three or more vascular factors tended to show higher performance compared to participants who suffered from one or two factors, but lower than healthy ones. This may be explained by the protective mechanisms that each participant may use in order to deal with their pathology. For instance, Fung [58] argued that the combination of physical activity and the intake of the appropriate medication in older adults with vascular risk factors helps them maintain their cognitive functions. On the same line, higher educational level also may function in a protective way in people suffering from vascular risk factors, as it provides a form of neural compensation and contributes to cognitive reserve [59,60], even when people deal with more than one vascular pathology. So, it may be assumed that people who suffer from many vascular risk factors could have other characteristics (e.g., high educational level) or could have adopted other strategies or mechanisms to deal with them, compared to people with one or two vascular risk factors, and these function in a compensatory way, contributing to the maintenance of their cognitive functions. Analyzing our sample, we came across the two most common combinations of three or more factors among participants. These were “smoking × lack of exercise × overweight” and “hypercholesterolemia × hypertension × lack of exercise”. More than half of those participants were of higher educational level, which possibly functioned in a protective way, confirming the above notion.

Additionally, there were some single effects that seem to contribute to binding processes but need further research. Specifically, there were indications that age-group slightly influenced binding processes. This is in agreement with other papers’ findings that age-related hippocampal dysfunction is related to worsened feature binding in WM [23]. Forsberg, Johnson and Logie [61] proposed that difference in feature binding between young and older adults may be attributed to the use of different cognitive strategies, suggesting different neural backgrounds [62]. On the same page, Allen, Atkinson and Nicholls [63] supported that strategic prioritization plays an important role in feature binding, as even if older adults have worsened WM in general, they preserve the ability to strategically direct their attention to more important items in WM, stressing the significance of cognitive strategies in feature binding.

Apart from the above, we also found a slight interaction effect of age-group and VRP. Aging is associated with the deterioration of vasculature [64]. This deterioration is highly related to cognitive and, especially, executive dysfunction [65]. However, it should be noted that, in this study, middle-aged adults were among the oldest age-group that participated. Furthermore, we noticed that by adding the VRP variable to our study, the effect of age on participants’ performance on FBT seemed to be almost nullified. This leads us to the conclusion that age functions as a descriptive indicator for underlying vascular disorganization and pathology. 

Another interesting observation that was made is the fact that even though a few participants were prescribed appropriate medication to deal with their vascular pathology, there did not seem to be any difference in their performance compared to participants who were not treated. This was not examined appropriately in this study, but it is something that needs to be examined in future studies.

At this point, it should be mentioned that the vast majority of participants belonged to the first level of VRP, meaning that they suffered from only one risk factor and especially lack of exercise. From those that suffered from three vascular risk factors, lack of exercise was also in the two most common combinations as already mentioned above. In addition, we also noticed that the interaction between smoking and lack of exercise played a role in binding processes. This is in agreement with papers suggesting that frequent, consistent exercise in combination with a healthy, nutritious diet can prevent the deleterious consequences of smoking and benefit cognitive function and reserve in healthy older adults, acting in a protective way, whereas discontinued exercise, poor nutrition and cigarette smoking are likely to raise the risk of cognitive decline [66]. Specifically, tobacco smoking, due to nicotine and its connection to a wide range of neurotransmitter systems, seems to affect a wide variety of cognitive domains and thus leads to faster cognitive decline [36,67]. In particular, nicotine alters hippocampal cholinergic functioning by activating hippocampal nicotinic acetylcholine receptors (nAChRs), which seem to play a role in the regulation of glutaminergic and GABAergic activity in the hippocampus [68,69]. Hippocampal cholinergic signaling is an important regulator of synaptic plasticity and, subsequently, of hippocampus-dependent learning and memory throughout the lifetime [69]. Particularly, this dysregulation of acetylcholine is also associated with the dysfunction of working memory and top-down regulation of attention in the dorsolateral prefrontal cortex [70]. On the same page, a cohort study that linked smoking with the risk of dementia concluded that even in heavy smokers, the cessation of smoking in early midlife may have positive effects on cognitive functions [71]. Furthermore, smoking affects cognitive function in an indirect way by exacerbating the damage caused by other vascular risk factors [72]. Particularly, when combined with other cardiovascular risk factors, it may lead to cardiac events and ischemia, and on a second level, it may contribute to endothelial damage and atherosclerosis [72,73,74] due to its metabolic effects (increased lipolysis) [72]. Thus, concerning our findings on the interaction of lack of exercise and the aforementioned data about nicotine, we can assume that smoking, when combined with other risk factors, indeed raises the danger for vascular pathology and, indirectly, cognitive impairment.

There is a vast number of studies highlighting the benefits of exercise on cognitive functions. Particularly, there are many interventions based on exercise designed to help people suffering from dementia or other neurodegenerative diseases (e.g., Parkinson’s), showing the importance of frequent physical activity on brain functions [75,76,77,78,79,80,81,82,83]. Erickson, Leckie and Weinstein [84] suggested that there is an association between physical activity and greater gray matter volume in the same regions that are associated with cardiorespiratory fitness, including the hippocampus and prefrontal cortex. Koščak Tivadar [85] mentioned that increased blood flow in the prefrontal cortex and parietal areas, in combination with the increased influence of the environment, subserves synaptogenesis and neurogenesis. Working memory, thinking and cognitive flexibility are the cognitive functions that are affected the most by physical activity [85].

This study has some limitations. The fact that information about each participant’s health was obtained only by a self-report questionnaire and not by any objective medical test may have had implications on the findings. Moreover, as this study was the first in which FBT was administered in its computerized form and in a remote way, additional cognitive tools should have been included in the battery for comparison. However, this was not the focus of this study. Additionally, the results were based on a convenience sample, which may have influenced them.

An interesting proposal for future research designs would be to administer this promising tool to other groups of people, such as older adults or people with neurodegenerative diseases, in order to examine its effectiveness and be able to extract norms. Subsequently, it would be of great interest to investigate further how this tool could be used widely in clinical practice, in order to detect early cognitive deficits in people suffering from any kind of vascular pathology and assist clinicians in the diagnostic process.

## 5. Conclusions

To conclude, there seems to be a common neural “situation” between vascular pathology and the binding processes of WM. Thus, although our results are not explicit, they manage to propose a new, alternative way to identify cognitive deficits in early phases, which needs further exploration.

## Figures and Tables

**Table 1:** Individual–demographic–clinical information for the sample of the study.

		Gender		Age Group		Educational Level (in Years)		
N = 229	Subgroups	Male	Female	Young Adults	Adults	Low (≤6 Years)	Medium (7–12 Years)	High (≥13 Years)
Vascular risk factors	Healthy	21	31	34	18	6	11	35
	1 factor	15	59	31	43	7	26	41
	2 factors	20	43	19	44	9	10	44
	≥3 factors	11	29	9	31	7	9	24
Hypercholesterolemia	Yes	19	43	8	54	9	11	42
	No	48	119	85	82	20	45	102
Hypertension	Yes	21	33	5	49	11	9	34
	No	46	129	88	87	18	47	110
Diabetes	Yes	4	9	3	10	0	4	9
	No	63	153	90	126	29	52	135
Overweight	Yes	19	48	22	45	6	18	43
	No	48	114	71	91	23	38	101
Smoking	Yes	12	40	19	33	8	12	32
	No	55	122	74	103	21	44	112
Exercise	Yes	51	88	51	88	14	33	92
	No	16	74	42	48	15	23	52

## Data Availability

The data presented in this study are available on request from the corresponding author.
